# Accurate and Rapid Differentiation of Acinetobacter baumannii Strains by Raman Spectroscopy: a Comparative Study

**DOI:** 10.1128/JCM.01744-16

**Published:** 2017-07-25

**Authors:** Meron Ghebremedhin, Rae Heitkamp, Shubha Yesupriya, Bradford Clay, Nicole J. Crane

**Affiliations:** a Regenerative Medicine Department, Naval Medical Research Center, Silver Spring, Maryland, USA; b Henry M. Jackson Foundation for the Advancement of Military Medicine, Bethesda, Maryland, USA; c Wound Infections Department, Walter Reed Army Institute of Research, Silver Spring, Maryland, USA; d Clinical Research Management, Hinckley, Ohio, USA; e bioMérieux, Inc., Instrumentation R&D, Hazelwood, Missouri, USA; f Department of Surgery, Walter Reed and Uniformed Services University of Health Sciences, Bethesda, Maryland, USA; Marquette University

**Keywords:** AST, Acinetobacter baumannii, DNA sequencing, hierarchical cluster analysis, Raman spectroscopy, mass spectrometry

## Abstract

In recent years, matrix-assisted laser desorption ionization–time of flight mass spectrometry (MALDI-TOF MS) has become the standard for routine bacterial species identification due to its rapidity and low costs for consumables compared to those of traditional DNA-based methods. However, it has been observed that strains of some bacterial species, such as Acinetobacter baumannii strains, cannot be reliably identified using mass spectrometry (MS). Raman spectroscopy is a rapid technique, as fast as MALDI-TOF, and has been shown to accurately identify bacterial strains and species. In this study, we compared hierarchical clustering results for MS, genomic, and antimicrobial susceptibility test data to hierarchical clustering results from Raman spectroscopic data for 31 A. baumannii clinical isolates labeled according to their pulsed-field gel electrophoresis data for strain differentiation. In addition to performing hierarchical cluster analysis (HCA), multiple chemometric methods of analysis, including principal-component analysis (PCA) and partial least-squares discriminant analysis (PLSDA), were performed on the MS and Raman spectral data, along with a variety of spectral preprocessing techniques for best discriminative results. Finally, simple HCA algorithms were performed on all of the data sets to explore the relationships between, and natural groupings of, the strains and to compare results for the four data sets. To obtain numerical comparison values of the clustering results, the external cluster evaluation criteria of the Rand index of the HCA dendrograms were calculated. With a Rand index value of 0.88, Raman spectroscopy outperformed the other techniques, including MS (with a Rand index value of 0.58).

## INTRODUCTION

The emergence of multidrug-resistant (MDR) nosocomial infections caused by bacteria such as Acinetobacter baumannii has become a global problem for both civilian and military populations. MDR infections can be problematic due to the small number of effective drugs for treatment (1). A rapid and accurate strain typing method becomes important in order to detect epidemiological outbreaks of nosocomial infections and provide appropriate and timely intervention in infection control (2). Traditional DNA-based methods of typing, such as multilocus sequence typing (MLST), microsatellite genotyping, amplification fragment length polymorphism (AFLP), DNA microsatellite typing, and pulsed-field gel electrophoresis (PFGE), can be used to obtain accurate and reliable results (3, 4). However, routine application of such methods has become impractical due to their high cost and time-consuming processes (4).

Matrix-assisted laser desorption ionization–time of flight mass spectrometry (MALDI-TOF MS) has become an ideal solution for overcoming these hurdles by providing rapid and accurate results and, as a result, has moved to the forefront of routine bacterial identification (5–7). However, some studies have shown the unsuitability of MALDI-TOF MS for strain-level discrimination in closely related bacterial species, including A. baumannii (4, 8–11). This inconsistency of MS in differentiating between closely related strains is hypothesized to arise primarily because the closely related species or strains express many similar, if not identical, proteins (12). This can result in multiple identical MS peaks, making discrimination between species or strains difficult.

In recent years, advances have been made in the application of Raman spectroscopy for bacterial identification. Raman spectroscopy is a rapid and noninvasive vibrational spectroscopic technique that yields molecular fingerprint information from biological samples such as bacteria. Numerous studies have successfully identified bacteria at the strain level by using Raman spectroscopy and surface-enhanced Raman spectroscopy (SERS) spectral databases of the microorganisms (13–25). In our previous work (25), Raman spectra of 42 bacterial samples that encompassed a wide range of bacterial species (10 different genera) were analyzed. Models were developed and successfully validated using a test set at the Gram and genus taxonomic levels. In this work, we developed hierarchical cluster analysis (HCA) models to differentiate between A. baumannii strains, with the goal of using Raman spectroscopy for strain-level identification in the future.

Although Raman spectroscopy has the capability of providing detailed molecular information by generating vast amounts of data, this can also be a drawback because the actual spectral interpretation is complex and requires robust data analysis and a comprehensive database (26). Moreover, fluorescence background can sometimes overwhelm Raman spectra, diminishing or hiding the Raman spectral peaks. Here, we evaluate the capability of Raman spectroscopy to reliably differentiate the A. baumannii strains responsible for clinical infections using hierarchical cluster analysis. Additionally, the HCA results for Raman spectral data are compared to the clustering results for three other data types, including data from mass spectrometry, DNA sequencing, and antimicrobial susceptibility testing (AST). For each resulting dendrogram, the strain type (based on PFGE type) is indicated on branches.

## RESULTS

**FIG 1:**
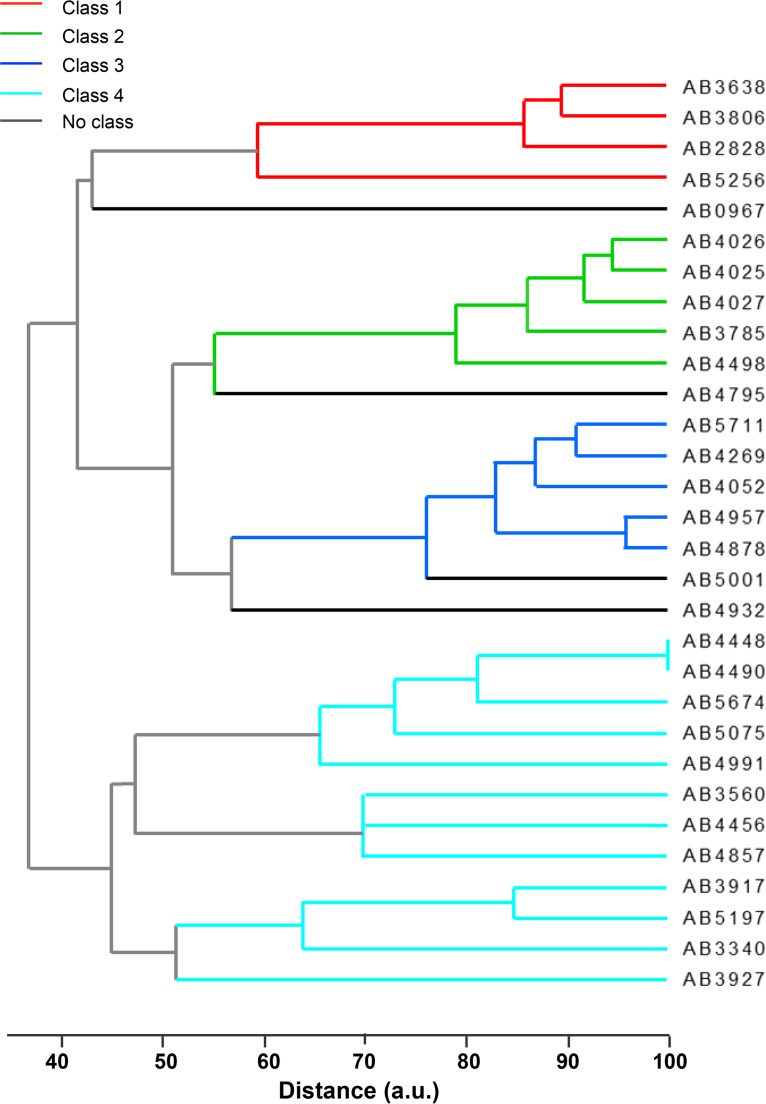
PFGE dendrogram of 30 A. baumannii clinical isolates clustered using unweighted pair group method with arithmetic mean (UPGMA) implemented in Bionumerics 7.5 software (Applied Maths, Sint-Martens-Latem, Belgium). Clades on the resulting dendrogram were separated into PFGE classes 1, 2, 3, and 4. Some strains could not be assigned to a PFGE class.

**FIG 2:**
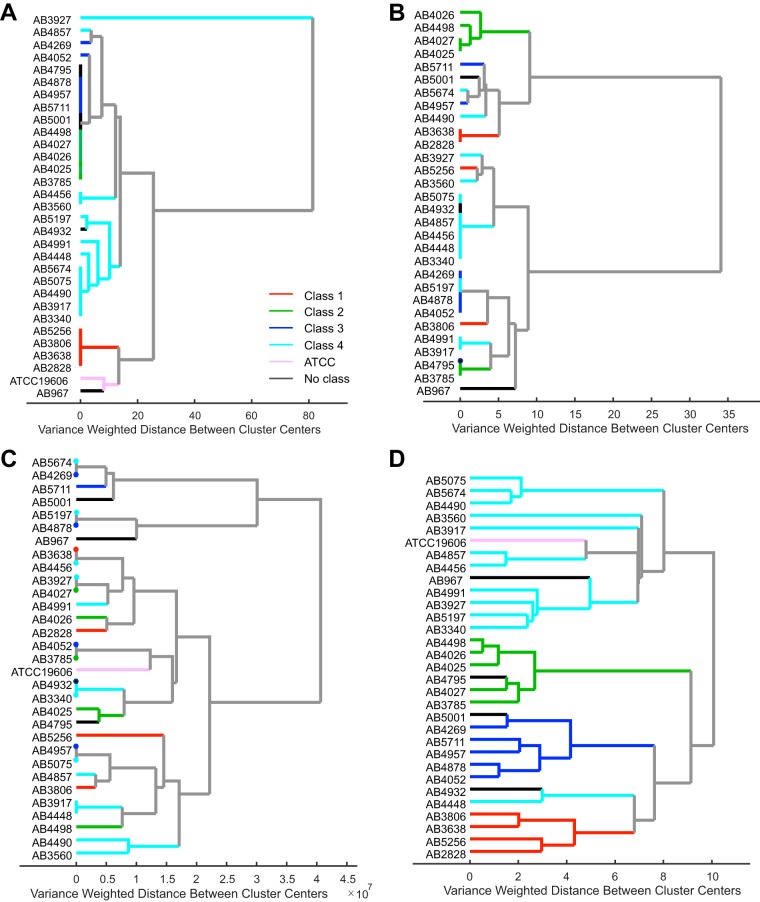
Hierarchical cluster analysis (HCA) results of A. baumannii clinical isolates using DNA sequences aligned via MAFFT (multiple alignment using fast Fourier transform) and numerically coded to perform HCA using Ward's linkage and Euclidean distance (A), antimicrobial susceptibility testing data using Ward's linkage and Euclidean distance (B), preprocessed MS data performed using Ward's linkage and Euclidean distance (C), and preprocessed Raman spectroscopic data after performing principal-component analysis (D). HCA was done using Ward's linkage and Mahalanobis distance. All dendrograms are colored according to their resulting strain type (based on PFGE type, indicated by classes 1, 2, 3, and 4).

Initially, a dendrogram was plotted using the PFGE banding pattern outcomes (Fig. 1). Clades on the resulting dendrogram were separated into PFGE classes 1, 2, 3, and 4. Some strains could not be assigned to a PFGE class. The PFGE classes were then utilized as class labels for the rest of the data sets. As demonstrated in Fig. 2, four dendrograms were plotted using DNA sequencing, AST, MS, and Raman spectral data sets of 31 A. baumannii strains.

In order to quantitatively compare the performance of the Raman spectral hierarchical clustering to the clustering of the other data types, external criteria of Rand index values were calculated using the *a priori* knowledge of ApaI digestion-PFGE classes (Fig. 1). The Rand index is defined as the number of pairs of objects that are either in the same groups in both partitions or in different groups in both partitions, divided by the total number of pairs of objects (27, 28). The value of the Rand index lies between 0 and 1, where 1 indicates that two partitions agree perfectly, and 0 indicates that the two partitions disagree completely (29). This method is useful in comparing cluster results with different numbers of clusters between the groups being compared, which is the case in the data sets reported here.

Due to the different types of data sets being analyzed here, it is expected that the hierarchical trees will demonstrate some variability in their structure. Therefore, the Rand index values were calculated by “cutting the dendrogram,” a method where the tree is scanned to detect the level possessing the optimal recovery of desired clusters (29). Each of the dendrograms displayed in Fig. 1 was individually evaluated, and a cutoff point was determined to reveal the inherent groupings of the data sets according to their PFGE classes. Those without a class label (strains AB967, AB4795, AB4932, AB5001, and ATCC 19606) were excluded for the Rand index calculations.

The dendrogram for the DNA sequences (Fig. 2A) matches fairly well with the PFGE classes with the exception of two misclustered isolates from class 4 and merging of isolates from classes 2 and 3 into one branch, giving a Rand index value of 0.78. AST successfully clustered class 2 isolates and the majority of class 4 isolates, giving a Rand index value of 0.70, while the rest of the isolates did not group well according to PFGE classes (Fig. 2B). The dendrogram for the MS data (Fig. 2C) shows the most disagreement with the PFGE classes, also resulting in the lowest Rand index value (0.58), compared to the rest of the data sets. Raman spectral data most accurately clustered the strains according to PFGE, forming four distinct clusters corresponding to the four PFGE classes, as depicted in Fig. 2D, and demonstrated a high Rand index value of 0.88. Leave-one-out cross-validation accuracy of Raman spectral clustering was calculated using the cutting the dendrogram method described above for each of the 26 models, which resulted in 92.3% accuracy for strain differentiation, with the misclustering of samples AB3560 and AB4448.

## DISCUSSION

Among bacterial strain typing methods, PFGE is considered to have the most discriminatory power in inferring relationships between strains and has been utilized as the gold standard for identifying microbial outbreaks (30–32). It provides high-resolution macrorestriction analysis, resolving microorganism identity using genomic data (32). Despite its advantages, however, PFGE is a time- and labor-intensive process with high consumable costs. Moreover, the technique's shortcomings include a lack of interlaboratory reproducibility and an inability to differentiate between bands nearly identical in size (32).

Therefore, many studies have explored alternative methods of microbial identification that are accurate, rapid, and cost-effective, such as MS and Raman spectroscopy (4, 10, 12–18, 33, 34). Other methods, such as DNA sequencing and AST, have also been used to differentiate among strains of microorganisms, including A. baumannii (35–37). AST, although not a primary bacterial typing method, was included in this study to examine its relationship with PFGE, Raman spectroscopy, and other typing methods. AST is implemented when antimicrobials cannot be predicted reliably based on knowledge of microbial identity (38); nonetheless, it has been shown to correlate with PFGE results in A. baumannii clinical isolates (36) and other bacterial species (35); therefore, it was added to this study for comparison.

In this study, Raman spectral data most accurately clustered A. baumannii strains according to their PFGE class via HCA, which was not only noticeable visually but was also reinforced by the high Rand index value. Similar results were observed by Maquelin et al. (18), where Raman spectra of a collection of well-characterized Acinetobacter species were analyzed via HCA and compared to HCA results of amplified fragment length polymorphism (AFLP) typing. Their findings, much like ours, showed highly similar groupings by both techniques. For the Raman data set, performance could be further improved with alternative spectral preprocessing and further exploration of more discriminative spectral regions. In our study, the agreement between PFGE and Raman spectroscopy results is not surprising; PFGE provides discrimination on the basis of differences in DNA sequences, and Raman spectroscopy provides discrimination on the basis entire cells, including DNA sequences.

In order to investigate the reasons behind successful clustering of Raman spectra, we examined the loading of the first few principal components (PCs) of the Raman spectra that accounted for the most variance in the data. The first PC, which accounted for 50.77% of variance, was predominantly composed of the region between 1,200^−1^ and 1,400 cm^−1^, with peaks at 1,254 cm^−1^ and 1,305 cm^−1^; these vibrational bands, associated with the amide III envelope, can be indicative of proteins (39). The second and third PCs, which accounted for 15.21% and 10.70% of variance, respectively, represented Raman spectral peaks at 979 cm^−1^, 1,129 cm^−1^, 1,378 cm^−1^, and 1,411 cm^−1^. The 979 cm^−1^ band, which was prominent in PC2, was observed in specific A. baumannii strains in some of our previous studies; thus, PC2 is attributed to the bacterial capsule phenotype (40). Three of the A. baumannii strains in this study (AB5075, AB5674, and AB4490) exhibit this unique 979 cm^−1^ band and, as a result, clustered distinctly with each other, as seen in Fig. 2D. The third PC (PC3) was composed of a mix of protein- and lipid-related vibrational bands; the amide III envelope and the 1,179 cm^−1^ band are typical protein vibrational bands, while the spectral peaks at 1,078^−1^ and 1,378 cm^−1^ are hallmarks of lipid vibrational bands, all of which can be associated with cell membrane components (41).

**FIG 3:**
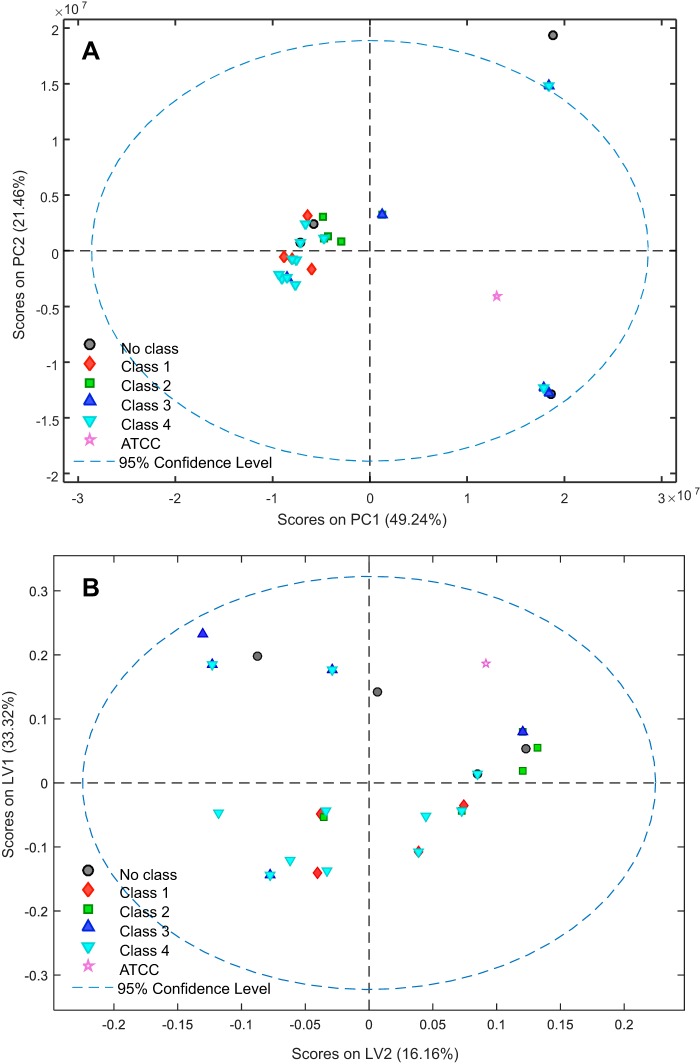
(A) PCA model of mass spectrometry data, preprocessed using a fourth-order Savitzky-Golay derivative and mean centering (spectral region used: 0 to 4,000 *m/z* values). (B) PLSDA model of mass spectrometry data, latent variable biplot of spectra preprocessed by normalizing to all variables and mean centering (spectral region used, 0 to 3,300 *m/z* values). Data are colored according to strain type (based on PFGE type indicated by classes 1, 2, 3, and 4).

While much success has been achieved in the application of MS to identify A. baumannii isolates at the species level (5–7, 33, 34), difficulties seem to arise with MS when it is applied for strain-level identification of A. baumannii isolates (4, 8–11). In this study, to further evaluate the discriminative capability of MS, multiple chemometric methods were implemented aside from HCA, such as PCA and PLSDA, along with various preprocessing methods (Fig. 3). However, no optimal separations were observed between the four PFGE classes. With the MS data, many of the samples from different PFGE classes show no clustering by class, further supporting the idea that MALDI-TOF MS has difficulty identifying closely related bacterial species and strains due to their close, if not identical, protein expressions (4, 12). This close relationship between MS spectra is also exhibited in the dendrogram, where many strains in the same cluster, but belonging to different PFGE classes (such as AB5674 and AB4269, AB3638, AB4456, etc.), show a straight line on the distance axis, which indicates that there is no linkage distance between them.

While MALDI-TOF is rapid and relatively easy, this report, along with other studies (4, 8, 10, 11), revealed that there are significant disadvantages for its application in closely related microorganisms. For example, in a study done by Rim et al. (10), dendrograms were generated by MS data for A. baumannii strains collected from an intensive care unit and were compared to a dendrogram generated using PFGE results. The study concluded that the dendrogram function of MALDI-TOF MS could not substitute for PFGE in MDR A. baumannii clonality analysis. Another study by Kishii and coworkers demonstrated only 72.4% accuracy for MALDI-TOF MS identification of Acinetobacter isolates from blood cultures (8). Finally, Alvarez-Buylla et al. concluded in a recent study that species within the genus Acinetobacter still require molecular techniques for identification (11).

In this study, however, we took one step further and compared MS spectral differentiation of strains with Raman spectroscopic differentiation of strains along with additional data types for A. baumannii isolates. To our knowledge, this the first study of this type to have been performed. The clear advantages of Raman spectroscopy shown here are that it is as rapid as MS and lower in cost while providing reliable and more highly discriminative power than MS for A. baumannii strain identification. Moreover, this study also demonstrates the advantages of Raman spectroscopy over both gene sequence and antimicrobial resistance data types for A. baumannii strain differentiation. The accuracy of the leave-one-out cross validation of the Raman spectral data model is an encouraging factor in improving and building on such models using additional data and validates using a separate test data set for future work. The enhanced performance of Raman spectroscopy for A. baumannii strain differentiation in this study may be attributed to the fact that the Raman spectra contain information about the whole cell, including cell morphology, rather than just genomic data.

In this study, hierarchical cluster analysis of Raman spectroscopic data, mass spectrometry, DNA sequence, and AST data sets of 31 A. baumannii clinical isolates was performed. As evidenced by the Rand indices of the different data sets, Raman spectroscopy more accurately differentiated among strains than the other typing methods. Furthermore, this study highlights the unreliability of MALDI-TOF MS, the current clinical standard for routine bacterial identification, in differentiating between closely related A. baumannii strains. This report suggests Raman spectroscopy as a promising new method for routine strain-level identification in A. baumannii clinical isolates. Future work will include developing and validating models using additional A. baumannii isolates and other MDR bacterial species using Raman spectroscopic data.

## MATERIALS AND METHODS

### Bacterial isolates.

30 clinical isolates of A. baumannii were obtained from the Wound Infections Department at Walter Reed Army Institute of Research. This set of isolates has been previously published (42), and represents a diverse sample of MDR A. baumannii isolates recovered from military personnel under treatment at Walter Reed Army Medical Center in Washington, DC. A reference strain of A. baumannii (ATCC 19606) was obtained from the ATCC (Manassas, VA, USA).

### Pulsed-field gel electrophoresis.

Isolate strain identity was initially determined via restriction fragment length polymorphism (RFLP) followed by pulsed-field gel electrophoresis (PFGE). Briefly, genomic DNA was digested by an ApaI restriction enzyme, and digested products were run on an agarose gel under an electric current to produce a DNA banding pattern. The banding pattern similarity coefficient was calculated using Dice's coefficient with 1% band-matching tolerance, and the results were clustered using unweighted pair group method with arithmetic mean (UPGMA) and implemented in Bionumerics 7.5 software (Applied Maths, Sint-Martens-Latem, Belgium).

### DNA sequencing.

Three genomic loci were sequenced for each isolate in the study. The name and primers for each locus are putative permease (5′-AAC ATG GGA TGG CTT GGT TTT-3′ and 5′-CAG ATC TAC CCG TGC CTT GAT AA-3′), glutamyl tRNA ligase (5′-CAA ACC GCA TAG GAA AGA AAA GA-3′ and 5′-CCT GAG AGG GAA TCA AAC TT-3′), and oxidoreductase (5′-CAA ACC GCA TAG GAA AGA AAA GA-3′ and 5′-AAG TCC GCC CAG GTC AGC-3′]. Primers were designed using Lasergene (DNASTAR, Inc., Madison, WI, USA) and synthesized by IDT (Integrated DNA Technologies, Coralville, IA, USA).

PCR was performed for each of the three loci in each isolate. The reactions were prepared using GoTaq Green master mix (Promega, Madison, WI, USA) in 50-μL reactions according to the manufacturer's instructions. The reactions were run on a DNA Engine Dyad thermal cycler (Bio-Rad, Hercules, CA, USA) under the following conditions: 95°C for 2 min, followed by 35 cycles of 94°C for 1 min, 45°C for 1 min, and 72°C for 1.5 min. Final extension at 72°C was done for 3.5 min. A final volume of 15 μl of amplified product was analyzed by agarose gel electrophoresis to confirm successful amplification. Reactions were purified and sequenced by Macrogen USA (Rockville, MD, USA). Resulting sequences were trimmed for quality using a Sequencher (Gene Codes, Ann Arbor, MI, USA).

### Antimicrobial susceptibility testing.

AST of isolates was performed on a BD Phoenix system (BD, Franklin Lakes, NJ, USA). The panels used for testing in this study were NMIC/ID-128 and NMIC/ID-132 (BD).

### Mass spectrometry.

A. baumannii isolates were received from the Naval Medical Research Center (NMRC) on blood agar slants (catalog number R060310; Remel, Lenexa, KS, USA). The isolates were grown on Trypticase soy agar (TSA) with sheep blood agar (catalog number R01201; Remel) for 18 to 24 h at 37°C in a 5% CO_2_ incubator. MALDI-TOF MS data were collected on a Vitek MS system (bioMérieux SA, Marcy l'Étoile, France). A colony of interest was sampled using a 1-μl inoculating loop. The collected sample was then smeared onto a MALDI slide (catalog number 410893; bioMérieux SA). This was done four times to make four spots using four different colonies per plate. A suspension of α-cyano-4-hydroxycinnamic acid (Vitek MS CHCA, bioMérieux SA) was overlaid onto each sample (approximately 1 μl dispensed by pipette). The matrix was allowed to dry before the slide was loaded into the mass spectrometer for analysis. Each group of 16 spots was tested along with a spot of Escherichia Coli (ATCC 8739), which served as a calibration target as well as a quality control indicator.

Mass spectra were generated in positive linear mode with a nitrogen laser (λ = 337 nm) operating with a pulse repetition frequency of 50 Hz. The ions were accelerated using a potential of 20 kV and an extraction delay time of 200 ns. For each sample, 100 spectra were averaged and obtained from different positions of the target spot, operating in conjunction with the Acquisition Station software. A spectral range of 2,000 to 20,000 Da was collected. Peak lists and intensities for each isolate were then submitted for data analysis.

### Raman spectroscopy.

Isolates were grown on LB agar plates for 48 h at 37°C in 5% CO_2_ and transferred into a 1.75-in.-width 0.38-in.-depth disposable plastic weigh dish covered with aluminum foil for spectral collection. A growth time of 48 h was used because it generated enough biomass to be transferred with a 10-μl inoculating loop and smeared on the disposable weigh dish to make nine different spots (∼3 mm in diameter each) to be measured by a particular Raman spectrometer. Raman spectra of the isolates were collected using a 785-nm Kaiser Rxn1 system (KOSI, Ann Arbor, MI, USA) equipped with a fiber-optic PhAT probe attachment with a 3-mm spot size lens. Each spectrum was the sum of 50 accumulations of 2 s and was collected for nine different spots of the same A. baumannii isolate; thus, nine Raman spectra were collected for each of the isolates.

### Data analysis.

All processing of data (aside from DNA sequence alignments) was performed in MATLAB using PLS Toolbox (Eigenvector Research, Inc., Wenatchee, WA, USA).

### DNA sequencing.

DNA sequences of the 31 A. baumannii isolates were aligned using multiple alignment using fast Fourier transform (MAFFT) (43). The sequences for each locus were aligned separately and then concatenated. The concatenated sequences were then numerically coded and imported into MATLAB to perform hierarchical cluster analysis (HCA).

### Antimicrobial susceptibility testing data.

Cluster analysis was also performed using the AST data for the 30 clinical isolates (excluding reference strain ATCC 19606 for lack of AST data). Data were numerically coded, and HCA was performed using Ward's linkage and Euclidean distance.

### Mass spectrometry data.

**FIG 4:**
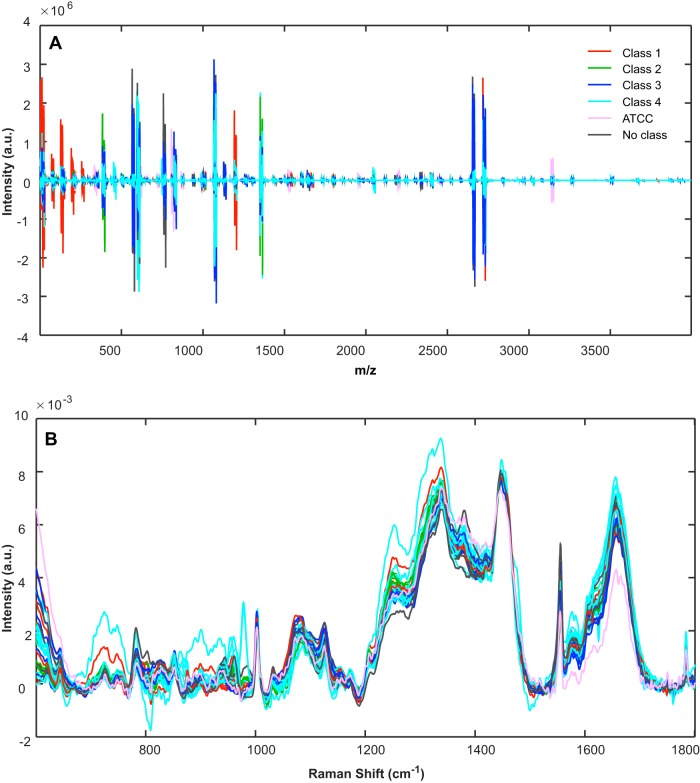
Overlaid, preprocessed spectra of 31 A. baumannii clinical isolates using MS data, preprocessed by taking the third-order derivative with a filter width of 15, followed by mean centering (A), and Raman spectroscopic data preprocessed by truncation of data between the 600 and 1,800 cm^−1^ Raman spectral region, baseline removal using a sixth-order polynomial, and normalization to the 1,445 cm^−1^ Raman spectral band (B). Spectra are colored according to their resulting strain type (based on PFGE type indicated by classes 1, 2, 3, and 4).

Four spectra per sample were collected. In order to account for spectral variability due to electronic variation in timing, a binning technique was implemented. Spectra were binned by identifying all the individual *x*-axis *m/z* values for each sample, finding the minimum and maximum values from this set, and building a mass axis of constant spacing with a desired input resolution (in this case, a resolution of two data points). Spectra were then averaged per sample and preprocessed by taking the third-order derivative with a filter width of 15 followed by mean centering (Fig. 4A). Finally, HCA was performed using Ward's linkage and Euclidean distance.

### Raman spectroscopy data.

Spectra were averaged per sample. Spectrum preprocessing included truncation of the Raman spectrum to a range of 600 to 1,800 cm^−1^, baseline removal using a sixth-order polynomial, and normalization to the 1,445 cm^−1^ Raman spectral band (Fig. 4B). The region between 900 and 1,540 cm^−1^ of the preprocessed data was then selected and mean centered before principal-component analysis (PCA) and hierarchical cluster analysis were performed. For HCA, nine principal components were used along with Ward's linkage and Mahalanobis distance. In order to validate this model, leave-one-out cross validation was carried out by performing PCA using 30 isolates and projecting the held-out test sample onto the PCA space. This was done 26 times (for the 26 samples with PFGE class labels). The first nine principal components of these models were used to create HCA models, along with Ward's linkage and Mahalanobis distance.
